# PMMA-Based Bone Cements and the Problem of Joint Arthroplasty Infections: Status and New Perspectives

**DOI:** 10.3390/ma12234002

**Published:** 2019-12-02

**Authors:** Alessandro Bistolfi, Riccardo Ferracini, Carlo Albanese, Enrica Vernè, Marta Miola

**Affiliations:** 1 AO Citta’ della Salute e della Scienza. CTO Hospital, Department of Orthopedics. Via Zuretti 29, 10126 Turin, Italy; abistolfi@cittadellasalute.to.it (A.B.); carlo.albanese@edu.unito.it (C.A.); 2 IRCCS Ospedale Policlinico San Martino, Department of Surgical Sciences and Integrated Diagnostics, University of Genova, Largo R. Benzi 10, 16132 Genova, Italy; riccardoferraciniweb@gmail.com; 3 Department of Applied Science and Technology, PolitoBIOMed Lab, Politecnico di Torino, C.so Duca Degli Abruzzi 24, 10129 Torino, Italy; enrica.verne@polito.it

**Keywords:** polymethyl methacrylate-based bone cement, additives, antibiotics, antibacterial, bioactive

## Abstract

Polymethyl methacrylate (PMMA)-based bone cement is a biomaterial that has been used over the last 50 years to stabilize hip and knee implants or as a bone filler. Although PMMA-based bone cement is widely used and allows a fast-primary fixation to the bone, it does not guarantee a mechanically and biologically stable interface with bone, and most of all it is prone to bacteria adhesion and infection development. In the 1970s, antibiotic-loaded bone cements were introduced to reduce the infection rate in arthroplasty; however, the efficiency of antibiotic-containing bone cement is still a debated issue. For these reasons, in recent years, the scientific community has investigated new approaches to impart antibacterial properties to PMMA bone cement. The aim of this review is to summarize the current status regarding antibiotic-loaded PMMA-based bone cements, fill the gap regarding the lack of data on antibacterial bone cement, and explore the progress of antibacterial bone cement formulations, focusing attention on the new perspectives. In particular, this review highlights the innovative study of composite bone cements containing inorganic antibacterial and bioactive phases, which are a fascinating alternative that can impart both osteointegration and antibacterial properties to PMMA-based bone cement.

## 1. Introduction

Bone cement, or PMMA (polymethyl methacrylate), is a polymer belonging to the category of acrylic resins, obtained from the mixture at room temperature of a monomer (methyl methacrylate, MMA) and a polymer (pre-polymerized polymethyl methacrylate particles) in the presence of an initiator, an activator, and a stabilizer [1]. PMMA-based bone cements are widely used in orthopedics, with two different functions: (1) to fix the joint arthroplasties to the bone (2), and to act as a temporary spacer for two-stage revision of septic, infected joint arthroplasties. However, they are inert materials that are unable to stimulate anchoring to bone tissue and are prone to bacterial contamination. A first strategy to reduce the bacteria adhesion to bone cement surfaces is to load PMMA-based bone cements with different antibiotics [2]. Nevertheless, the efficacy of antibiotic-loaded bone cements (ALBCs) is still debated; in particular, the doubts of the researchers concern the method to introduce a drug, its amount and release, and the mechanical performance of the materials [2,3,4,5,6]. Moreover, the use of ALBCs is connected to the issue of antibiotic resistance [2,6]. For this reason, the research community started to investigate alternative strategies to develop antibiotic-free bone cements with antibacterial properties.

The aim of this review is to provide a comprehensive overview of the different approaches adopted to impart antibacterial properties to PMMA-based bone cement, starting from antibiotic-loaded bone cement to innovative formulations, highlighting the ongoing issues in this area.

## 2. History and Use of PMMA-Based Bone Cement

In 1933, Dr. Otto Rohm patented the PMMA product plexiglass, and in 1936 Kulzer discovered that the dough formed by mixing PMMA powder and MMA hardens when benzoyl peroxide is added. The first clinical use of this PMMA mixture to close cranial defects in monkeys occurred in 1936, while the era of modern PMMA bone cements started in 1943 (patented by Degussa and Kulzer).

**Figure 1:**
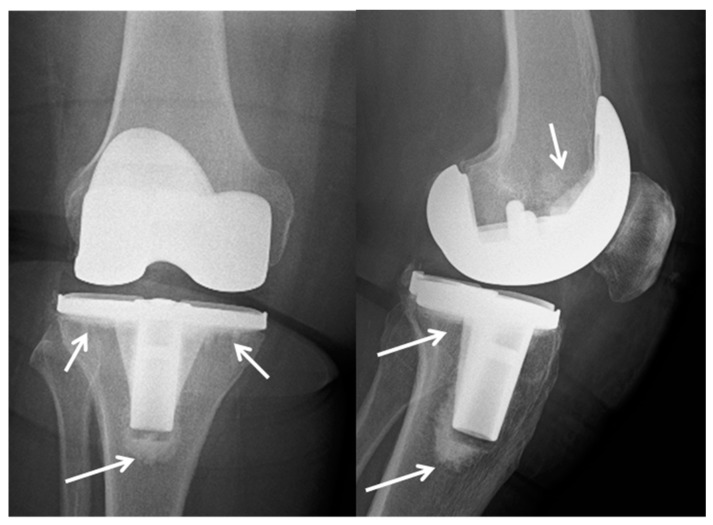
X-ray of a cemented total knee arthroplasty in the right knee of a 73-year-old woman at six months follow up. The arrows indicate the cement.

The first use of this technology was for dental fixatives and fixtures, but in 1958 it was also adopted in orthopedics by Dr. John Charnley. Finally, in the mid-1970′s, the Food and Drug Administration (FDA) approved the use of bone cement technology in the United States [7]. Figure 1 shows an example of a cemented total knee arthroplasty in the right knee of a 73-year-old woman at six months follow up. The arrows indicate the cement.

**Figure 2:**
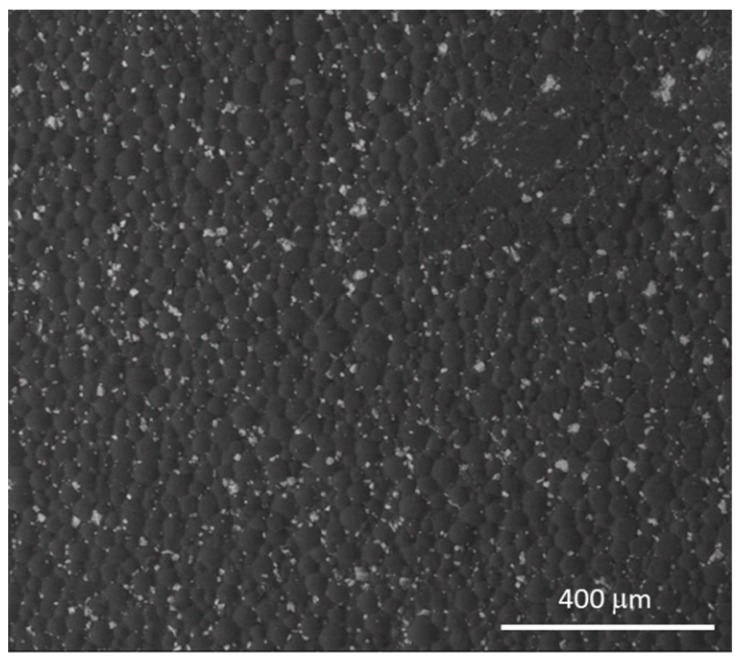
Scanning electron microscopy (SEM) image of a PMMA-based bone cement, containing zirconia as a radio-opaque agent.

Bone cement is produced directly in the operating room during surgery by mixing two components: (1) Liquid: mainly composed of MMA, with the addition of *N*,*N*-dimethyl-*p*-toluidine, which promotes the polymerization process, and hydroquinone as a stabilizer to prevent self-curing of the monomer in the liquid during storage. (2) Powder: mainly composed of PMMA particles, with the addition of benzoyl peroxide, which triggers polymerization when mixing occurs, along with zirconia (ZrO_2_, Figure 2) or barium sulphate (BaSO_4_) as radiopaque agents. Table 1 presents the compositions of the most used PMMA-based bone cements.

The cement preparation process can be divided into four phases: (1) mixing phase, (2) waiting phase, (3) working phase, (4) hardening or setting phase.

The polymerization reaction is exothermic and the polymerization temperature is influenced by various factors, including the physical composition of the PMMA, thickness of the cement, and endosteal and periosteal circulatory conditions. 

PMMA is characterized by having good biocompatibility with human tissues, but has lower mechanical value than bone tissue, including: (a) less resistance to compression; (b) less resistance to fatigue; (c) less tensile strength.

Finally, other problems connected to PMMA-based bone cement are the weak mechanical bond to bone tissue and the lack of antimicrobial properties; bacteria can adhere to the cement–bone interface, creating an inflammatory process.

## 3. Postsurgical Orthopedic Infections

The main orthopedic infection that affects bone is called osteomyelitis. Out of all osteomyelitis cases, infections arising from replacement arthroplasty surgery are particularly significant and difficult to treat; these are called periprosthetic joint infections (PJI). The extent of PJI in primary hip and knee procedures is estimated to be in the range 0.5–3%, while in primary arthroplasties the rates remain low [8].

**Figure 3:**
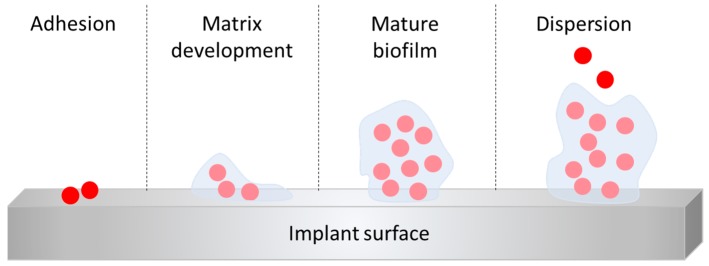
Schematic representation of biofilm formation steps.

The presence of a foreign body, such as an orthopedic implant, has been shown to significantly increase susceptibility to infection. Promoting early osteoblast adhesion is essential to prevent bacterial colonization of components and promote osteointegration. If other cells adhere first, such as osteoclasts, this induces the recognition of the implant as a foreign body and induces the immune response against them. It is worse if they were bacteria present first. Bacterial colonization of the implant is the decisive step in implant-related infections and depends on the ability of bacteria to adhere to a given surface. The adhesion involves the classical physicochemical forces (Van der Waals attraction, electrostatic charges, gravitational forces, hydrophobic interactions) and specialized adhesion molecules of the bacteria [9]. In vitro, colonization appears within a few hours and slime production occurs within several days, depending on experimental conditions. It is now apparent that biofilm formation is a rather complex, genetically driven process, mediated by the number of bacteria-derived signaling molecules, also known as “quorum sensing (QS) molecules” or autoinducers. The basic steps of biofilm formation (Figure 3) are quite similar among the bacteria species: bacteria attach to a surface by means of specialized adhesion molecules, then signalling molecules are released, which in turn drive the biofilm formation by inducing the production of the extracellular matrix (extracellular polymer substances, EPS) and the name-giving (in some instances visible) film or slime, and also by altering bacteria-inherent features and properties, for example the loss of flagella. The bacteria are then embedded in the extracellular matrix, which is the most conspicuous feature of the biofilm, yielding a well-organized bacterial community. Depletion of nutrients or waste product accumulation in biofilms causes micro-organisms to enter a slow or non-growth (stationary) state, making them up to 1000 times more resistant to most antimicrobial agents than their free-living counterparts and allowing them to persist for months or years [10]. Moreover, the presence of biofilm is difficult to diagnose, since cells are protected by the biofilm layer, which can impede culturing of the bacteria.

## 4. Classification, Symptoms Diagnosis, and Treatment of Joint Periprosthetic Infections (PJIs)

Several classifications of periprosthetic infections have been proposed, according to different factors: (1) the time between the intervention and the diagnosis of PJI (Tsukayama classification); (2) the method of contagion; (3) the etiological agent involved (Table 2). Epidemiological data regarding the incidence of PJI show a percentage of infection of 1.5–2% in total hip arthroplasty (THA) and 2.5–5% in total knee arthroplasty (TKA). The overall risk of a PJI is relatively low between 0.5% and 3% (Table 3). Despite these data, given the increasing number of patients subjected to total joint arthroplasty, a significant increase in PJIs is expected in the coming years. The classic symptomatology in the case of PJI includes: (a) temperature; (b) erythema, swelling, and local pain at the level of the surgical site; (c) serous, blood, and purulent fluids leaking from the surgical wound; (d) pain, even at rest. However, in chronic forms, the most common and often unique symptom is pain [11,12].

The diagnosis of a PJI is sometimes challenging, and the diagnostic criteria are still debated in the orthopedic community, as reported in the Second International Consensus Meeting on Musculoskeletal Infections. Serum ESR (erythrocyte sedimentation rate) and CRP (C-reactive protein) are good indicators but they are poorly specific for infection and are recommended in first-line screening tests. Their use is recommended in combination with other markers such as white blood cells (WBC), IL-6, procalcitonin (PCT), and D-dimer. Other markers showed potential but did not demonstrate validity; these include advanced glycation endproducts, thiobarbituric acid reactives, lipopolysaccharide binding proteins, preserpin, and soluble intercellular adhesion molecules. Aspiration of the peri-prosthetic synovial fluid may allow identifying the etiologic agent, performing sn antibiogram, and setting a specific antibiotic therapy. Aspiration also allows the analysis of synovial fluid biomarkers such as WBC count, polymorphonuclear neutrophilis count, alpha-defensin, and cytochines (IL-6, leukocyte esterase), which can be helpful for the detection of a PJI. Radiography, CT (computed tomography), and MRI (magnetic resonance imaging) may show mobilization of the implant or fluid accumulation between the implant and the bone with soft tissue involvement (MRI) and are helpful to indicate a possible PJI; however, they are not specific for PJi diagnosis [6,7,13,14,15]. It is important to evaluate the outcome trends following the intervention. Scintigraphy is very sensitive, but not very specific if a three-phase technique is used with Tecnetium-99; it is more sensitive and specific if a technique based on labeled leukocytes is used. Next-generation sequencing (NGS) is a new molecular technology potentially able to revolutionize the clinical microbiology laboratory [16]. It is based on the collection of non-Sanger-based high-throughput DNA sequencing methods, which allow obtaining large amounts of data, at low cost and in a short time. This technique is one of the emerging diagnostic tests that also include the analysis of interleukin-6, alpha-defensin, and serum D-dimer [16].

Several strategies for the treatment of PJI have been reported [17,18,19,20], including: (1) debridement, surgical irrigation, and replacement of small prosthetic components and subsequent antibiotic therapy; (2) one-stage, prosthetic replacement and subsequent antibiotic therapy; (3) two-stage prosthetic replacement and subsequent antibiotic therapy (gold standard); (4) removal without replanting (arthrodesis); (5) amputation; (6) chronic therapy, i.e., antibiotic treatment without replacement or removal of the prosthetic components. 

## 5. Cement with Antibacterial Activity: Antibiotic-Loaded Bone Cements and Innovative Solutions

One of the major uses of the cements is serving as a temporary spacer during two-stage revisions. In this case, an antibacterial activity is useful in the treatment of infections. Moreover, a PMMA-based cement able to limit bacterial adhesion could be useful for first-stage joint arthroplasty implant. Therefore, many strategies have been suggested to confer some sort of bactericidal or antibacterial activity to the cement, in particular by adding or mixing antimicrobial substances into the cement.

The first section summarizes the basic principles of antibiotic-loaded PMMA and evidences the improvement that the new approaches have provided to antibiotic release. The following sections report the state of the art of antibacterial, antibiotic-free PMMA bone cement. Even if at present the literature on this topic is limited, a survey is necessary since, on the basis of the authors’ knowledge, it was never carried out. Table 4 reports the types of antibiotic-free PMMA-based cement with antibacterial properties tested so far.

### 5.1. Antibiotic-Loaded Bone Cement

The use of antibiotic bone cement has assumed an increasingly important role in the prophylaxis and treatment of PJIs [41,42]. Antibiotic cement (antibiotic-loaded bone cement, ALBC) consists of a bone cement loaded with an antibiotic. The choice of the right antibiotic, the way in which it is mixed with the cement, the antibiotic amount, and the time and the modalities of release of the drug to the surrounding tissues are of paramount importance in its use [2,7,43,44,45,46,47]. The choice of the antibiotic plays an important role in the success of the antiseptic effect of the antibiotic cement. In fact, the antibiotic must have some important characteristics: (a) a broad antibacterial spectrum (including Gram-positive and Gram-negative bacteria); (b) a low percentage of resistant species; (c) soluble in water; (d) non-allergenic; (e) thermostable (the exothermic polymerization reaction of the cement must not cause structural and functional changes).

Based on the characteristics mentioned above, the antibiotics that are most frequently mixed with cement are: (1) gentamicin; (2) tobramycin; (3) erythromycin; (4) vancomycin. Table 5 lists the most used and investigated antibiotics and their combinations.

**Figure 4:**
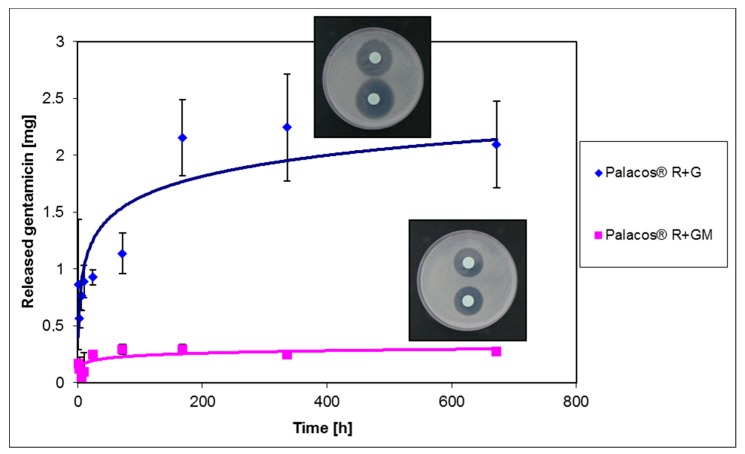
Release trend and inhibition halo images of gentamicin for commercial antibiotic-containing cement (Palacos^®^ R + G) and cement with gentamicin manually added (Palacos^®^ R + GM).

ALBC releases the antibiotic locally and directly to the site of infection. This allows higher local concentrations to be maintained compared to those obtained with intravenous administration. These concentrations exceed the limit of sensitivity for pathogens, allowing their elimination before they can develop a protective biofilm. The dosage of the antibiotic mixed with cement allows the ALBC to be divided into 2 categories: high doses of antibiotics (atb) (atb >2 g for 40 g of cement) and low doses (atb <2 g in 40 g of cement). The rationale for the choice between the two categories is the implant period: high-dose ALBC are usually adopted in cement spacers or beads (temporary implants), while low-dose ALBC are used for prophylaxis. The use of ALBC containing more than 2 g of antibiotic (for 40 g of cement) is harmful for the mechanical properties of cement and can compromise the stability of the implant itself. For this reason, the use of cements loaded with more than 2 g of antibiotic is only recommended for spacers. The release of the antibiotic is strictly time-dependent and biphasic, with a peak in the early hours, followed by a slow and progressive decrease over the following months or years. The initial release depends on the roughness of the surface: the greater the roughness, the greater the area of release; a week after the intervention, a linear dependence between the porosity of the cement and the release of the antibiotic was demonstrated. Some studies have also shown how the release of cement also depends on the type of cement used [48]. To achieve a broader spectrum of action and efficacy it is possible to combine two or more antibiotics, exploiting their synergistic effect [47,49,50]. Comparison between spacers loaded with the combination of gentamicin and vancomycin and spacers loaded with gentamicin alone has shown the higher antibacterial efficacy of the former. Finally, the antibiotic can be industrially blended into the pre-polymerized PMMA or can be manually added by a surgeon at the surgical site [17,51]. Figure 4 reports the trend of cumulative release of gentamicin-containing Palacos^®^ R + G (commercial sample) and Palacos^®^ R + GM (sample with gentamicin manually (M) added), together with the respective images of inhibition zones [16]. As can be noticed, in this study the authors observed a similar release antibiotic trend for both cement formulations, but commercially-loaded cements released a higher amount of gentamicin than Palacos^®^ R + GM and showed a larger inhibition zone (i.e., greater antibacterial effect) towards *S. aureus* strain. The best blending process has been thoroughly investigated in literature; however, at present, the best mixing method remains under debate [3,4,5,52].

Recent studies proposed different PMMA-based bone cement formulations in order to control and improve the release of antibiotics [53,54,55,56,57]. As an example, Chen et al. [53] suggested the incorporation of gelatin spheres (up to 600 μm) into antibiotic-releasing PMMA bone cement (Tecres S.P.A). Gelatin introduction allows the creation of an interconnective porous structure that promotes antibiotic release, as a result of an improved channel network in the PMMA matrix, which facilitates the drug diffusion into the liquid.

The use of nanocarries, such as silica nanoparticles [55,56], alginate, polyhydroxybutyratehydroxyvalerate (PHBV), and ethylcellulose and stearic acid (SA) nanocapsules [54] was also studied, evidencing, in general, an improvement of the antibacterial effect, also in terms of prolonged time [55,56], without degrading the cement characteristics such as mechanical properties and setting time.

Shen et al. [57] incorporated an antibiotic (gentamicin) in hollow nanostructured titanium dioxide nanotubes, which were incorporated in a PMMA matrix to create nano diffusion networks and promote the drug’s release. They evidenced that titanium dioxide nanotubes enhanced the release of the antibiotic, since more than 50% of gentamicin was released in two months, in comparison with pristine cements that released only 5% of the drug.

### 5.2. Silver-Loaded Bone Cements

Silver is a metal that has often been associated with the medical industry because it was used as a bactericide before the discovery of antibiotics. This metal shows low toxicity but can be harmful if inhaled, if ingested, or with prolonged contact, causing a disease known as argyria. Silver releases ions that interact with the thiol groups of proteins, leading to the production of reactive oxygen species (ROS) that damage components of bacterial cells and viruses, determining alterations of the permeability of cell membranes, inhibition of enzymatic activities and DNA synthesis, and interruption of intracellular signal transduction. Silver can be used as an antimicrobial agent to coat first implant prostheses or spacers and can be utilized in various forms, such as powders, thin films, nanomaterials, nanocomposite coatings, and nanoparticles. For silver coatings, the reduction of pathogens is around 90% [3,5,21,22].

The literature proposes different forms of cement loaded with silver:

1. PMMA bone cement (Coripharm) loaded with metallic silver particles with sizes ranging 5–50 nm (nanosilver);
2. PMMA encapsulating silver nanoparticles (about 5 nm) capped with oleic acid;
3. PMMA (BIOLOS3^®^)/Ag_2_O particles (0.5%, 1%, and 2% *w*/*w*) bone cements;
4. PMMA cement (VersaBond) loaded with 0.1%, 0.5%, and 1% (*w*/*w*) high-porosity silver ranging 2–10 mm.

Studies have shown the good antimicrobial effect of silver-loaded bone cements. Volker et al. [21] demonstrated that nanosilver bone cement inhibited the proliferation of *S. epidermidis*, methicillin-resistant *S. epidermidis* (MRSE), and methicillin-resistant *S. aureus* (MRSA) in vitro, without affecting the material’s compatibility towards mouse fibroblasts and human osteoblasts. Prokopovich et al. [22] encapsulated oleic-acid-capped silver nanoparticles into PMMA-based bone cement at various ratios. They reported an antimicrobial activity against several strains, including MRSA, at Ag nanoparticles concentrations as low as 0.05% (*w*/*w*); moreover, biocompatibility tests on osteoblast cells did not show cytotoxic behavior and cements maintained their mechanical properties. Moreover, Prokopovich et al. [23] proposed another formulation containing silver–tiopronin nanoparticles; the silver–tiopronin nanoparticles were produced with different dimensions (about 5 nm and 11 nm in diameters) and inserted into bone cement at various ratios (1%, 0.5%, and 0.1% *w*/*w*). The performed analyses showed that cements containing small nanoparticles did not produce an antibacterial effect, while cements loaded with larger nanoparticles were able to reduce the contamination of MRSA in vitro, even at the lowest concentration (0.1 *w*/*w*), without affecting either biocompatibility or mechanical properties. Slane et al. [24] proposed commercial bone cement containing commercial Ag nanoparticles (30–50 nm, surface-functionalized with polyvinylpyrrolidone) at different ratios (0.25%, 0.5%, and 1.0% *w*/*w*) and demonstrated that Ag-nanoparticle-loaded samples were able to reduce the formation of *S. aureus* and *S. epidermidis* biofilms on their surface, even if they showed no effect towards planktonic cells. A good antimicrobial effect was also demonstrated by Wekwejt et al. [25], introducing commercial Ag nanoparticles (50 nm, 1.5% and 3% *w*/*w*) into Cemex formulations. Samples showed the inhibition of adhesion and growth of five different bacterial strains and displayed good cytocompatibility.

However, in vivo tests performed by Moojen et al. [26] showed that silver nanoparticles containing bone cement predominantly exhibited an antimicrobial effect at the direct cement surface, while in vivo tests on rabbit model showed that this cement is not effective in prevention of methicillin-sensitive *Staphylococcal* infection, since the metallic particles are not released from the cement and need to be ionized before they exhibit their antimicrobial activity. Therefore, this material showed an antimicrobial effect only at the direct cement surface and not in the surrounding bone tissue.

Alt et al. [27], recently, performed a clinical study on 12 patients to assess the safety of silver-loaded gentamicin-PMMA spacers. In this study, silver nanoparticles (from 5 to 50 nm in diameter) were used; however, silver nanoparticles tended to aggregate and form microparticles of 2 to 5 µm in diameter, which were introduced into the PMMA spacers. Silver concentration was evaluated in blood, urine, and drainage fluids; moreover, a histopathological estimation of the periprosthetic membrane around the spacers was carried out. The analyses evidenced the safety of silver-containing cement; nevertheless, efficacy studies in terms of the cement ability to reduce the infection rate must be performed.

### 5.3. PMMA Bone Cements Containing Other Antibacterial Inorganic Agents

A recent study performed by Russo et al. [28] investigated the antibacterial effect of a commercial bone cement (Smartset Hv Bone Cement—DePuy) loaded with gold nanoparticles (10–20 nm in diameter). Different concentration of Au nanoparticles (0.25% *w*/*w*, 0.5% *w*/*w*, and 1% *w*/*w*) were added to the solid phase of the cement in order to impart antibacterial properties. The obtained results showed a reduction of biofilm formation (methicillin-resistant *Staphylococcus aureus* (MRSA) and *Pseudomonas aeruginosa*) for cement containing 1% of Au nanoparticles; however, the inclusion of a nanoparticles amount greater than 0.25% induced a slight reduction of mechanical properties.

The antimicrobial effect of copper nanoparticles (1.5% and 3% *w*/*w*) was also investigated by Wekwejt et al. [25], showing a high bactericidal effect towards *Staphylococcus aureus*, *Staphylococcus epidermidis*, *Enterococcus faecalis*, *Enterobacter cloacae*, and *Pseudomonas aeruginosa*, along with antibiofilm properties but a slight cytotoxicity towards dental pulp stem cells.

Finally, Sathya et al. [29] developed PMMA-based nanocomposites by incorporating CuO nanoparticles, cetyltrimethylammonium bromide (CTAB)-capped CuO, and ZnO (singly and in combination). The study evidenced the efficacy of all formulations against *Staphylococcus aureus* and *Escherichia coli* strains; furthermore, a cytocompatibility analysis, performed using the L6 myoblast cell line, showed that cements containing 0.1% (w/v) of nanoparticles were not toxic. In particular, the CuO-containing formulation was more effective against *E. coli*, while samples containing CTAB-capped CuO and ZnO were very effective against *S. aureus*. Moreover, the authors evidenced that the combination of nanoparticles improved the antibacterial activity of the nanocomposites against both the investigated bacterial strains.

The antibacterial effect of graphene oxide nanosheets (average size of 400 nm) dispersed in PMMA-based cement was also investigated [30]. Zapata et al. developed a composite bone cement containing chitosan and graphene oxide (0.3% *w*/*w*) and evidenced a good antimicrobial effect towards *E. coli*, without affecting the cytocompatibility. 

The incorporation of magnesium particles (100–150 μm, 0.1, 0.2, 0.4, or 0.8 g of Mg mixed with 2.6 g PMMA powder) into PMMA-based bone cement was also evaluated [31]. This study demonstrated a good in vitro antimicrobial effect of the composites towards *S. aureus* and *E. coli*; moreover, in vitro and in vivo studies evidenced a strong bone–implant interface, the promotion of osteoblasts adhesion, spreading, and proliferation, and endothelial cell angiogenesis.

### 5.4. Bone Cements Containing Organic Antibacterial Agents

Organic agents with antimicrobial effects have also been used as fillers to impart antibacterial properties to PMMA [32,33,34,35,36]. For example, Perni et al. [32] encapsulated paraben nanoparticles in PMMA-based bone cement and verified that nanoparticles at concentrations of 7% *w*/*w* prevent the growth of different pathogens, such as *Staphylococcus aureus*, methicillin-resistant *S. aureus*, *Staphylococcus epidermidis*, and *Acinetobacter baumannii*. Moreover, bone cement containing paraben nanoparticles maintained the compression strength, and no evidence of cytotoxic effect was observed on osteoblast cells (MC-3T3). Another study by Deb et al. [33] reported the effect of the inclusion of an antibacterial quaternary amine monomer (QAMA) (quaternized ethylene glycoldimethacrylate piperazine octyl ammonium iodide, containing a polymerizable group) into a commercial PMMA-based cement. The inclusion of QAMA did not modify the cement’s curing parameters and the mechanical properties in terms of tensile and compressive strength, and imparted an intrinsic radio-opacity. Besides, cements containing 15% of QAMA were able to inhibit the growth of E. coli and were cytocompatible towards a human osteosarcoma cell line. Finally, Shi et al. [34] synthesized PMMA-based bone cement containing chitosan nanoparticles (CS NP) and quaternary ammonium chitosan-derivative nanoparticles (QCS NP), eventually in combination with gentamicin. This study showed the ability of both CS NP and QCS NP-containing cements to inhibit the growth of *S. aureus* and *S. epidermidis*, using the LIVE/DEAD BacLight bacterial viability kits and fluorescence microscopy. Moreover, bone cements loaded with QSC NP showed 10^−3^ fold viable bacterial cell decrease. Finally, the insertion of chitosan-based nanoparticles enhanced the antibacterial effect of gentamicin-loaded cements. A similar study was proposed by Wang et al. [35], who developed a PMMA-based cement containing quaternized chitosan (*N*-(2-hydroxy)propyl-3-trimethylammonium chitosan chloride-based hydrogels loaded with nanosized hydroxyapatite and demonstrated an excellent in vitro bacteriostatic effect of the cement against *E. coli* and *S. aureus*.

The in vivo performance of a quaternized chitosan (hydroxypropyltrimethyl ammonium chloride chitosan, HACC)-loaded PMMA bone cement was investigated by Tan et al. [36] in a New Zealand rabbit model. This study evidenced the ability of the HACC-loaded bone cement to inhibit the adhesion and proliferation of an antibiotic-resistant *S. epidermis* strain, evidencing a good in vivo antibacterial activity of the proposed cement.

### 5.5. Bone Cements with both Bioactive and Antibacterial Phases

A different way to limit the bacterial adhesion on implant surfaces is to improve the adhesion and proliferation of osteogenic cells rather than the bacteria rate for the surface colonization [58], promoting a stable chemical bond with bone tissue. For this purpose, different bioactive fillers, such as bioactive glasses and hydroxyapatite, have been used in the literature [59,60,61]. However, an effective reduction of bacterial colonization on the surface of the bioactive bone cements has not been demonstrated. For this reason, some researchers have investigated the possibility of developing composite bone cements containing both a bioactive phase and antibacterial one [62,63,64]. The proposed formulations seem to be effective in reducing bacterial growth and promoting osteointegration, but often the addition of more phases has a negative effect on mechanical properties, and this approach seems to present some concerns regarding industrial and clinical feasibility. Moreover, the majority of the studies use antibiotics as the antibacterial agent, thus avoiding the problem of resistant bacterial strain development. Recently, some authors have developed new composite bone cements containing a single inorganic phase that is contemporaneously bioactive and antibacterial [37,38,39,40].

**Figure 5:**
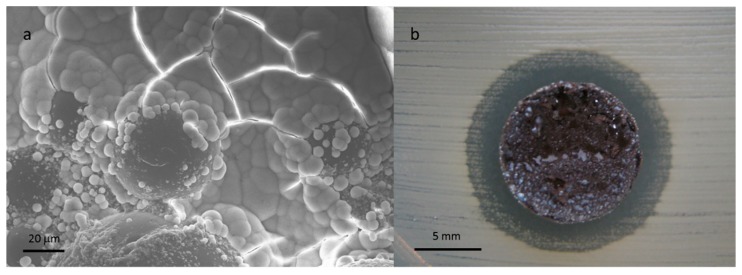
Hydroxyapatite precipitation on copper-doped glass containing composite bone cement (**a**). Antibacterial effect towards *S. aureus* (inhibition halo test) of a composite cement containing silver-doped glass (**b**).

The inorganic filler consisted of bioactive glass powders doped with antibacterial ions (e.g., Ag^+^, Cu^++^), which were inserted in different commercial PMMA-based formulations with different viscosities. The optimization of the particle grain size, the methods for incorporation of the antibacterial ions in the glass network (ion exchange process versus melt and quenching technique), and the glass amount allow the mechanical properties of the pristine cement and the biocompatibility to be maintained, and at the same time confer bioactive and antibacterial properties to the polymeric matrix (Figure 5) towards different strains. The obtained results represent a promising starting point for developing antibiotic-free and multifunctional materials for orthopedic prostheses fixation, temporary prostheses, and spinal surgery.

## 6. Conclusions and Future Perspectives

This review provides an overview on the problems connected to the use of PMMA-based bone cement, in particular the cement contamination by bacteria. Currently, the unique clinically adopted solution is the use of PMMA-laden bone cement; even if the effectiveness of antibiotic-loaded cement remains a debated issue, the improvement of PJIs treatment with local drug release continues, and new research concepts concern the encapsulation of antibiotics into micro- or nanocapsules. However, the development of multiresistant bacteria strains has stimulated the scientific community to investigate new approaches to impart antibacterial properties to this material. The use of inorganic antibacterial agents looks promising; in particular, the use of bone cement loaded with Ag nanoparticles seems to impart a good antimicrobial effect in vitro, as well as towards antibiotic-resistant cells. Nevertheless, further studies have to be performed to verify the in vivo performance of the proposed materials. Bone cements containing organic antibacterial agents also need further in vivo investigation. The combination of an antibacterial phase (in particular an inorganic agent) and a bioactive one seems to be an efficient and attractive alternative, since it contemporaneously allows the bond with bone tissue and limits the bacteria adhesion and proliferation. In this case, it must be taken into account that the mechanical properties of the obtained composite have to be carefully checked, together with the in vivo performance.

## 7. Patents

E. Verne’, M. Miola, S. Ferraris, A. Masse’, A. Bistolfi, M. Crova, G. Maina. Composite Bone Cements with a PMMA Matrix, Containing Bioactive Antibacterial Glasses or Glass-Ceramics. Patent specification EP2451493A2, WO2011004355A2, 13 January 2011, WO2011004355A3, 13 October 2011.

## Figures and Tables

**Table 1:** Composition of polymethyl methacrylate (PMMA)-based bone cement.

Component	Constituent
**Solid**	Polymethyl methacrylate (alone or in combination with other polymers, such as polymethacrylate or polystyrene)
	Zirconium dioxide or barium sulfate, as a radio-opaque agent
	Benzoyl peroxide, as an initiator
	Colorant (e.g., E141)
**Liquid**	Methyl methacrylate
	*N*,*N*-dimethyl-*p*-toluidine, as an accelerator
	Hydroquinone, as a stabilizer
	Colorant (e.g., E141)

**Table 2:** The classification of periprosthetic infections and involved pathogens.

The Classification of Periprosthetic Infections		
Time between the Intervention and the Diagnosis of PJI (Tsukayama Classification):	Method of Contagion:	Etiological Agent Involved:
Positivity of only intra-operative culture; Early post-operative infections, develop within 30 days of surgery; Late or chronic post-operative infections, develop after 30 days from surgery; Late acute haematogenic infections.	Intra-operative contamination; Post-operative direct contamination (inoculation of microorganisms through the surgical wound); Hematogenous contamination.	*Staphylococcus epidermidis* (36%); *Staphylococcus aureus* (29%); *Batteri anaerobi* (5%); *Enterococcus faecalis* (4%); *Escherichia coli* (3%); *Pseudomonas spp* (2%); Other pathogens (5%).

**Table 3:** Risk factors for a periprosthetic joint infections (PJIs).

Risk Factors for a PJI		
(1) General:	(2) Local:	(3) Specific:
Site of the surgery (knee: 2.5–5%, hip: 1.5–2%) Type of the surgery; Obesity; Malnutrition; Smoking; Alcoholism; Female sex; Old age; Skin infections; Treatment with corticosteroids; Immunodeficiencies; Concurrent diseases; Neoplastic diseases.	Localized sepsis (septic outbreaks in other districts); Peripheral vasculopathies with reduced oxygen supply; Previous surgical procedures at the same location; Previous joint infiltrations in the same site; Cutaneous fragility; Presence of post-operative hematoma.	Duration of the surgical procedure; Number of people in the operating room; Level of surgeon experience; Non-articular infections.

**Table 4:** Antimicrobial agents used to develop antibiotic-free antibacterial PMMA-based bone cement and in vitro, in vivo, and clinical tests.

Antibacterial Agent	In Vitro Test	In Vivo Test	Clinical Test	Refs.
Ag nanoparticles (5–50 nm)	*Staphylococcus aureus* (*Methicillin-Resistant Staphylococcus aureus* MRSA) *S. epidermidis* (*Methicillin-Resistant Staphylococcus epidermidis* MRSE) *S. epidermidis*			[21]
Oleic acid-capped Ag nanoparticles (about 5 nm)	*Staphylococcus aureus* *Staphylococcus aureus* *Staphylococcus epidermidis* *Acinetobacter baumannii*			[22]
Ag–tiopronin nanoparticles (5–11 nm)	*Staphylococcus aureus* (MRSA)			[23]
Ag nanoparticles functionalized with polyvinylpyrrolidone (30–50 nm)	*Staphylococcus aureus* *Staphylococcus epidermidis*			[24]
Ag nanoparticles (50 nm)	*Staphylococcus aureus* *Staphylococcus epidermidis* *Enterococcus faecalis* *Enterobacter cloacae* *Pseudomonas aeruginosa*			[25]
Ag nanoparticles (5–50 nm)		rabbit model		[26]
Ag nanoparticles (5–50 nm)			12 patients	[27]
Gold nanoparticles (10–20 nm)	*Staphylococcus aureus* (MRSA) *Pseudomonas aeruginosa*			[28]
Copper nanoparticles	*Staphylococcus aureus* *Staphylococcus epidermidis* *Enterococcus faecalis* *Enterobactera cloacae* *Pseudomonas aeruginosa*			[25]
CuO nanoparticles (20–50 nm), (CTAB)-capped CuO (10–30 nm)	*Staphylococcus aureus* *Escherichia coli*			[29]
ZnO nanoparticles (20–120 nm)	*Staphylococcus aureus* *Escherichia coli*			[29]
Graphene oxide nanosheets (400 nm)	*Escherichia coli*			[30]
Magnesium particles (100–150 nm)	*Staphylococcus aureus* *Escherichia coli*			[31]
Paraben nanoparticles	*Staphylococcus aureus* (MRSA) *Staphylococcus epidermidis* *Acinetobacter baumannii*			[32]
Antibacterial quaternary amine monomer (QAMA)	*Escherichia coli*			[33]
Chitosan nanoparticles Quaternized chitosan (220–284 nm)	*Escherichia coli* *Staphylococcus aureus* *Staphylococcus epidermidis*	Rabbit model		[34,35,36]
Bioactive glasses (<20 μm) doped with Ag ^+^ or Cu^2 +^ )	*Staphylococcus aureus* *Escherichia coli* *Bacillus* *Candida albicans*			[37,38,39,40]

**Table 5:** The most used antibiotics and their combination in antibiotic-loaded bone cements (ALBC).

Antibiotic	Efficacy	Combination
Gentamicin	Good	Vancomycin, Clindamycin
Vancomycin	Good	Gentamicin, Tobramycin
Tobramycin	Good	Vancomycin
Clindamycin	Good	Gentamicin
Ciprofloxacin	Good	
Cephalosporine	Moderate	Gentamicin
Tetracycline	Poor	
